# Early impact of X‐ and Y‐chromosome variations (XXX, XXY, XYY) on social communication and social emotional development in 1–2‐year‐old children

**DOI:** 10.1002/ajmg.a.62720

**Published:** 2022-03-14

**Authors:** Nienke Bouw, Hanna Swaab, Nicole Tartaglia, Anna C. Jansen, Sophie van Rijn

**Affiliations:** ^1^ Clinical Neurodevelopmental Sciences Leiden University Leiden the Netherlands; ^2^ Leiden Institute for Brain and Cognition Leiden the Netherlands; ^3^ Developmental Pediatrics, Children's Hospital Colorado University of Colorado School of Medicine Aurora Colorado USA; ^4^ Department of Pediatrics University of Colorado School of Medicine Aurora Colorado USA; ^5^ Pediatric Neurology Unit, Department of Pediatrics UZ Brussel Jette Belgium; ^6^ Neurogenetics Research Group Vrije Universiteit Brussel Brussels Belgium

**Keywords:** early social communication, Klinefelter syndrome, sex chromosome trisomies, social emotional functioning, trisomy X

## Abstract

Sex chromosome trisomies (SCTs) are characterized by an extra X‐ or Y‐chromosome (XXX, XXY, XYY). The present study aims to investigate early signs of social communication and social emotional development in very young children with SCT. Thirty‐four children with SCT (aged 12–24 months) were included in this study, as well as 31 age‐matched controls. Social communication was measured with structured behavior observations according to the Early Social Communication Scales, and social emotional developmental level with the Bayley Social Emotional parental questionnaire. Recruitment and assessment took place in the Netherlands and in the United States. On average, 12–24‐month old children with SCT showed difficulties with early social communication, more so in responding to others as compared to initiating social communications. During social interactions, children with SCT made less frequent eye contact, compared to controls. Also, difficulties in acquiring social emotional milestones were found in 1‐year old children with SCT, with 44% of the children having social emotional vulnerabilities in the borderline or extremely low range, compared to typically developing children. In this cohort, no significant predictive effects of karyotype‐subtype (XXX, XXY, XYY) were found. Already from a very early age, SCT can be associated with increased risk for vulnerabilities in adaptive social functioning. These findings suggest that SCT impact the maturation of the social brain already from an early age, and stress the importance of early monitoring and (preventive) support early social development in young children with SCT.

## 1 INTRODUCTION

Sex chromosome trisomies (SCTs) are specific genetic conditions that may serve as naturalistic “at risk” models of neurodevelopment, because they are associated with increased risk for neurobehavioral difficulties and psychopathology (Reiss et al., 2000). Because such genetic conditions can be diagnosed in the prenatal period or in infancy, this allows for the prospective study of developmental pathways toward specific vulnerabilities in children, adolescents, and adults with SCT. SCT is characterized by an extra X‐ or Y‐chromosome compared to the typical karyotype of 46,XX in girls and 46,XY in boys. Prevalence estimates of SCT vary from 1:650 to 1:1000 (Boyd et al., 2011). Knowledge about the impact of SCT on the neurocognitive and neurobehavioral phenotype is growing, and it is known that a disproportional high percentage of genes on the X‐chromosome play a role in human cognition and brain development (Zechner et al., 2001). There is evidence suggesting that an extra X‐ or Y‐chromosome convergently impact the maturation of a distributed and interactive network of cortical and subcortical components underlying adaptive social cognitive functioning, often described as the “social brain” (Hong & Reiss, 2014; Raznahan et al., 2016). Anatomical brain structures related to social functioning that seem to be affected by SCT include the posterior insula, the anterior cingulate, the medial prefrontal cortex, the superior temporal sulcus, and the orbitofrontal cortex (Raznahan et al., 2016).

Social brain alterations in SCT fit with the increased risk for deficiencies in the processing of social information and with difficulties in social emotional development, like problems in understanding social emotional information, and in showing social adaptive behavior during interactions with others, that have been observed in school‐aged children, adolescents, and adults with SCT. Although the phenotype of SCT is variable with some not having marked symptoms while others are more affected, studies that evaluated several domains of social adaptive functioning in individuals with SCT found social vulnerabilities including shyness, social immaturity, difficulties in forming adequate interpersonal relationships, increased levels of social anxiety and social impulsivity, and impairments in underlying social cognitive mechanisms (see for reviews: Freilinger et al., 2018; Tartaglia, Cordeiro, et al., 2010; Ross et al., 2012; Urbanus, van Rijn, & Swaab, 2020). The severity of social behavioral and social cognitive impairments in school‐aged children, adolescents, and adults with SCT is illustrated by an increased level of clinical diagnoses of Autism Spectrum Disorder (ASD). Average percentages of ASD classifications across studies vary from 15% (range 10.8%–20%) in individuals with 47,XXX; 18% (range 10%–27%) in individuals with 47,XXY; to 30% (range 19%–43%) in individuals with 47,XYY (Van Rijn, 2019).

In order to identify early markers of “at risk” social development and related social behavioral vulnerabilities later in life, it is important to study the impact of SCT on the social behavioral phenotype very early in life. The first study on social behavioral characteristics in children with SCT aged 1–5 years found social behavioral problems already at this age (Urbanus, Swaab, et al., 2020). Since social difficulties have a major impact on a broad range of areas of development, including language acquisition, school readiness, peer acceptance, and risk and resilience for developmental psychopathology, early identification of compromised social development in young children with SCT can help understand developmental outcome and finding targets for preventive intervention (Rao et al., 2008).

Early social development is marked by the growing ability to co‐ordinate eye contact and to engage in reciprocal social emotional interactions (Soto‐Icaza et al., 2015). Already in the first hours of life, infants prefer to look at faces that engage in eye contact (Farroni et al., 2002). Making eye contact with others is a basic biological mechanism, essential for social communication, as the visual information in eyes are important sources of information used to understand communicative goals and emotional states of others (Senju & Johnson, 2009). Thereafter, young children develop specific communication skills underlying socially adaptive behavior, in which the following elements are of particular importance: following eye gaze and conventional gestures of others in order to achieve shared attention to an object or event (i.e., joint attention), the ability to communicate own beliefs and desires and to react to the desires of others, and being part of reciprocal social interactions. These specific communication skills involve the use of eye contact and serve to hold and coordinate attention between interactive social partners (Mundy, 2003/2013). In naturalistic daily life settings, social communication skills are linked to significant accomplishments of broad social emotional functioning: they shape the capacity of the child to engage with others, to comprehend emotional expressions of others, and to elaborate upon a range of feelings in social interactions (Bayley, 2006).

As the first years of life is a period in which the social brain network rapidly matures and specializes, this period serves as a key period to acquire social emotional developmental milestones (Grossmann & Johnson, 2007). Therefore, it is especially important to focus on social behavioral abilities during that stage of early childhood as a means of identifying early markers of an “at risk” social development. The current study aims to provide in that by investigating the impact of SCT on the early communicative and social emotional phenotype in a group of children, aged 12–24 months.

The present study may have specific benefit to the clinical care of individuals with SCT. As SCT is being increasingly identified during pregnancy with recent technical advances of noninvasive prenatal screening (i.e., the noninvasive prenatal screening test [NIPT]), the population of infants with prenatal diagnosis of SCT is rapidly growing (Tartaglia et al., 2020). The study of very young children with SCT supports the identification of children with SCT at risk for neurobehavioral difficulties and psychopathology later on in life and will give insight in potential targets for monitoring, early prevention and intervention.

Taken together, the present study aims to investigate the impact of SCT on structured observations of early social communication and parental report of social emotional development in 1‐year‐old children. In addition to these main research questions, predictive effects of karyotype‐subtype (XXX vs. XXY vs. XYY) on early communicative skills and social emotional skills were studied. Based on the relevance of the X‐ and Y‐chromosome for the development of neural networks supportive of adaptive social functioning, we hypothesized that 1‐year old children with SCT might show less well developed social emotional and communicative skills, compared to a control sample.

## 2 METHODS

### 2.1 Participants

The present study is part of a larger ongoing project (the TRIXY Early Childhood Study – Leiden, the Netherlands), which includes children with SCT and nonclinical controls. A group of 34 children with SCT aged 12–24 months was included in this study (*M*_age_ = 1.39, *SD* = 0.36), as well as 31 age‐matched controls (13 boys; *M*_age_ = 1.53, *SD* = 0.28). Mean age did not significantly differ between groups (*t* (63) = 1.69, *p* = 0.096). The SCT group consisted of 6 girls with 47,XXX (17.6%), 20 boys with 47,XXY (58.8%), and 8 boys with 47,XYY (23.5%). Gender distribution differed between the SCT and control group (χ^2^ (1) = 11.37, *p* = 0.001). Recruitment and assessment took place on two research sites: the Trisomy of the X and Y chromosomes (TRIXY) Expert Center in the Netherlands, and the eXtraordinary Kids Clinic in Developmental Pediatrics at Children's Hospital Colorado in the United States. Children in the SCT group were recruited with the help of clinical genetics and pediatrics departments (from the Netherlands, Dutch speaking parts of Belgium and the United States), as well as through patient‐advocacy groups and social media postings. One girl with XXX, five boys with XXY, and three boys with XYY were recruited and assessed in the Netherlands. Five girls with XXX, 15 boys with XXY, and 5 boys with XYY were recruited and assessed in the United States. Karyotype distribution did not differ between the two research sites (χ^2^ (2) = 0.82, *p* = 0.664). Thirty‐two children (94.1%) were diagnosed prenatally, and two children postnatally (5.9%; one girl with XXX, one boy with XXY). Eleven out of 20 boys with 47,XXY had received testosterone treatment (55%).

The diagnosis of SCT was defined by trisomy in at least 80% of the cells, which was confirmed by standard karyotyping. For the SCT group, recruitment strategy was assessed, and three subgroups were identified: (1) “active prospective follow‐up,” which included families who were actively followed after prenatal diagnosis (67.6% of the SCT group); (2) “information seeking parents,” which included families who were actively looking for more information about SCT without having specific concerns about the behavior of their child (26.5% of the SCT group); and (3) “clinically referred cases,” which included families seeking professional help based on specific concerns about their child's development (5.9% of the SCT group). The control group was recruited from the western part of the Netherlands and approached with information brochures about the study. All participants were Dutch or English speaking, had normal or corrected‐to‐normal vision, and did not have a history of traumatic brain injury or hearing loss. For ethical reasons, children in the control group were not subjected to genetic screening, as these children were meant to be a representation of the general population. As the prevalence of SCT is ~1 in 1000, the risk of having one or more children with SCT in the control group was considered minimal and acceptable.

Parental education and age of the primary caregiver were assessed. Parental education was assessed according to the criteria of Hollingshead (Hollingshead, 1975). Scores of this scale include: 0 (no formal education), 1 (less than seventh grade), 2 (junior high school), 3 (partial high school), 4 (high school graduate), 5 (partial college or specialized training), 6 (standard college/university graduation), and 7 (graduate/professional training). Ninety‐eight percent of all parents indicated that their child has a second caregiver. If two parents were available, level of education was averaged over both parents. A Pearson χ^2^ test was performed to investigate possible differences in parental education distribution between the SCT and control group. No difference was found (χ^2^ (8) = 12.04, *p* = 0.149), indicating equal parental education in the SCT group (*M* = 5.96, *SD* = 0.88), and the control group (*M* = 5.47, *SD* = 1.32).

### 2.2 Measurements

#### 2.2.1 Background measures: Level of cognitive and language development

In order to measure general cognitive functioning and language development, the Bayley‐III (composite score of the cognitive and language subscale; Bayley, 2006) was administered in the original English version and the translated Dutch version (Bayley‐III‐NL; Steenis et al., 2012). This test aims to measure cognitive and language skills in children from 1 to 42 months of age.

#### 2.2.2 Early social communication: Early Social Communication Scales

The Early Social Communication Scales (ESCS; Mundy, 2003/2013) is a systematic behavior observation of a structured 20‐min play situation, designed to measure key early social communication skills that are usually acquired in the first 30 months of life. Following the procedures described by Mundy (2003/2013) the behavior ratings of specific defined responses during fixed time intervals were scored by trained independent raters. Raters were not involved in the assessment, and blind to the child's group membership and karyotype. Three distinct and mutually exclusive social communicative subscales were scored based on the videotaped session: joint attention (JA), behavioral requests (BR), and social interaction (SI). A distinction was made between initiating social communication and responding to social communication, resulting in six domains of early social communication. Variables of interest were the total scores on the six communicative subscales in addition to the frequency of eye contacts of the child during three of the ESCS domains (Initiating Joint Attention, Initiating Behavioral Requests, and Responding to Social Interactions). See Table 1 for a description of the nonverbal communicative behaviors within the six subscales of early social communication.

**TABLE 1:** Description of early social communication coded behavior during social interactions (based on Mundy, 2003/2013)

Joint attention		
Initiating Joint Attention	Eye contact	Child makes eye contact with examiner while manipulating or touching an inactive mechanical toy
	Alternate	Child alternates looking at active mechanical toy or a toy in their hand and the examiner's eyes
	Point	Child extends index finger toward toy within reach or to part of the room (e.g., posters)
	Show	Child extends toy toward the examiner's face
Responding to Joint Attention	Look	Child turns head and eyes in the direction of the examiner's pointing gesture, or to the appropriate area of a book

Behavioral requests		
Initiating Behavioral Requests	Eye contact	Child makes eye contact with examiner after a toy is removed from the child
	Reach	Child extends arm toward an out of reach toy
	Appeal	Child reaches to toys which are out of reach and makes eye contact with the examiner
	Point	Child points to toys that are out of reach
	Give	Child pushes toy toward examiner or holds an object out toward the examiner's hand or body
Responding to Behavioral Requests		Child responds to gestural and verbal requests of the examiner

Social interactions		
Initiating Social Interactions	Initiates turn‐taking	Child initiates game by rolling ball or car to the examiner
	Tease	Child engages in a prohibited activity (e.g., throwing toys from table) while smiling at the examiner
Responding to Social Interactions	Eye contact	Child makes eye contact with examiner after examiner has tickled the child
	Act	Child vocalizes or bangs the table or child reaches to examiner after the examiner has tickled the child
	Appeal	Child reaches to examiner and makes eye contact after being tickled
	Turn taking	Child takes turns with examiner throwing the ball or rolling the car
	Response to invitation	Child correctly places toys on or toward the examiners' head

#### 2.2.3 Social–emotional development: Bayley SE

The Bayley Social Emotional Questionnaire (Bayley SE; Bayley, 2006) consists of 21 or 24 items (dependent on the age of the child), and measures the acquisition of functional social–emotional milestones in naturalistic settings that broadly represent social–emotional patterns and developmental accomplishments. The Bayley SE questionnaire was administrated in the original English version (Bayley, 2006), and the translated Dutch version (Bayley SE‐NL; Van Baar et al., 2014). The items of the Bayley SE assess the attainment of age‐related milestones of the child, namely the ability to engage and use various emotions, expressions and experiences, as well as the comprehension of a range of social and emotional signals and to understand and react to a feelings of others with words, gestures, or imitations. Examples of items are: “Shows you that he or she understands your actions or gestures by making an appropriate gesture in return (e.g., make funny face back at you, looks at something you point to, stops doing something when you shake your head and use a firm voice to say ‘No!!’ or smiles and does more of something when you nod with a big smile and say ‘Yes!’)” and “Uses many consecutive actions in a back‐and‐forth way to show you what he or she wants or to have fun with you (e.g., smiles, reaches out for a hug, and, when you hug, takes your hat, puts it on his or her head, and smiles proudly OR takes your hand, leads you to the refrigerator, tugs on the handle, and, after you open it, points to something he or she likes, such as food, a bottle of juice, or milk).”

The primary caregiver rates his/her child on a 5‐point Likert scale ranging from 1 “none of the time” to 5 “all of the time.” Raw scores were used to compare the children with SCT and the controls. Based on the guidelines of the Bayley 3rd Edition Manual, the total raw scores of the Bayley SE were converted to a composite score, ranging from 55 to 145 with a mean of 100 and standard deviation (*SD*) of 15 points in the norm population. The composite scores were labeled as being in the average range (composite score > 90), the “Borderline/monitoring” range (composite score 70–89), and the “Extremely low/at risk” range (composite score < 70; Weiss et al., 2010). The Bayley SE has high internal consistency and test–retest reliability (Weiss et al., 2010), and the original validation study demonstrated that the Bayley SE distinguishes significantly between clinical groups including children with genetic syndromes or developmental disorders (Bayley, 2006).

### 2.3 Study procedures

**FIGURE 1:**
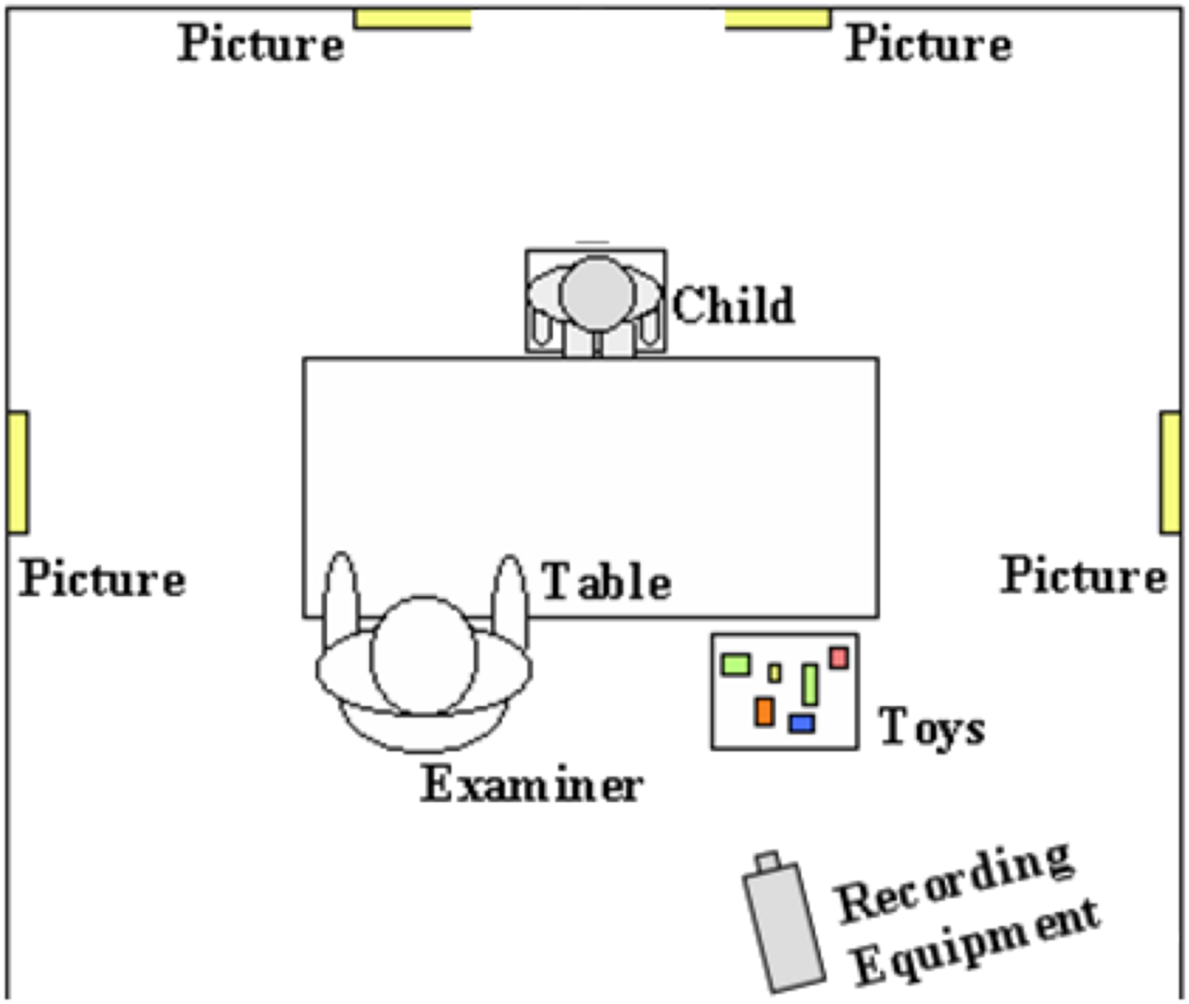
Setup assessment room ESCS administration (adapted from Mundy, 2003/2013)

Signed informed consent was obtained from the parents of all participating children, according to the declaration of Helsinki. This study was approved by the Ethical Committee of Leiden University Medical Center, the Netherlands, and the Colorado Multiple Institutional Review Board (COMIRB) in Colorado. Assessment took place at different sites (Colorado and the Netherlands) either in a quiet room at the University or at home. To standardize the testing environment, the testing setup and research protocols were identical on all sites. Researchers from Leiden University were responsible for project and data‐management (i.e., training and supervision of researchers processing and scoring of data). Administration of the ESCS always took place after administration of the Bayley‐III in order to prevent familiarity differences to interfere with the test scores. During the ESCS, the child was seated at a table across from a familiar examiner (see Figure 1 for the setup of the assessment room). Verbal interactions were kept to a minimum during the ESCS. The 20‐min structured assessment was videotaped, with full face view of the child and profile view of the experimenter. The parent questionnaire was completed by the primary caregiver of the child, either in Dutch or in English.

### 2.4 Statistical analyses

Data were analyzed using the Statistical Package for the Social Sciences (SPSS), version 25. Independent *t* tests and MANOVAs were carried out to test outcome differences between gender (boys vs. girls) and between research sites (the United States vs. the Netherlands). Independent *t* tests were used to test for differences between the SCT and control group for cognitive and language development. Multivariate analyses of variance (MANOVAs) were used to test differences on early social communication, with the scores on the six subscales of the ESCS (IJA, RJA, IBR, RBR, ISI, RSI) as dependent variables and research groups (SCT, control) as independent variable. Independent *t* tests were used to test differences on social emotional functioning, with the scores on Bayley SE as dependent variable and research groups (SCT, control) as independent variable. Multivariate and univariate analyses of covariance (ANCOVA and MANCOVA) were used to control for global cognitive and language level. To test SCT versus control differences in eye contact, three separate independent *t* tests were used with number of eye contacts on three subscales of the ESCS (IJA, IBR, RSI) as dependent variables. Pearson's correlation analyses were used to test the association between early social communication and daily life social emotional development in the SCT group. Linear regression with dummy coding was used to test for the effect of karyotype‐subtest and recruitment strategy on the scores on Bayley SE and ESCS. Level of significance was set at *p* < 0.05, 2‐tailed. Effect sizes were calculated with Cohen's *d* or partial *η* ^2^ when applicable.

## 3 RESULTS

### 3.1 Comparison between gender and research sites

No differences were found between for social communication on the six subscales of the ESCS (*F* (6, 26) = 1.34, *p* = 0.275), and social emotional functioning measured with the Bayley SE (*t* (29) = −1.95, *p* = 0.061) between control boys and girls. Therefore, data between boys and girls were collapsed across gender groups. Also, no differences between the research sites (the Netherlands and the United States) were found for social communication (*F* (6, 26) = 1.90, *p* = 0.120), and social emotional development (*t* (32) = −0.94, *p* = 0.355). Based on this, all SCT data were collapsed across sites.

### 3.2 Background measures: Cognitive and language development

The Bayley‐III is successfully completed by 64 children. Global cognitive development did not differ between the SCT (*M* = 99.85, *SD* = 13.26) and the control group (*M* = 99.71, *SD* = 13.98; *t* (62) = 0.41, *p* = 0.968). However, language development did differ between the SCT (*M* = 95.94, *SD* = 15.83) and the control group (*M* = 110.19, *SD* = 13.32; *t* (62) = −3.89, *p* <0.001).

### 3.3 Structured behavior observations of early social communication

#### Data quality

The ESCS was successfully completed by 63 children (two children are not able to complete the task). Although all children received at least 14 out of 18 trials of the active wind‐up toys and hand‐operated toys, some trials of four children are not administrated (e.g., because the child was crying) or are excluded due to technical aspects (e.g., experimenter obscured camera angle). Therefore, for these children the mean value of the coded social behaviors on other trials was used in place of these missing data (=mean substitution; Kang, 2013). Inter‐rater reliability was measured based on a subsample of 10 participants and showed an intraclass correlation coefficient (ICC) of 0.84–0.96 (for the ESCS domains collapsed together) which is considered excellent reliability (Cicchetti & Sparrow, 1981).

#### Early social communication and age

Within the SCT group, social communication was positively correlated with age on domains of Initiating Behavioral Requests, Responding to Joint Attention, and Responding to Social Interaction, indicating better social communicative skills in older children. No correlation with age was found for the other three ESCS domains. See Table 2 for exact Pearson's *r* and *p* values.

**TABLE 2:** Early social communication in children with SCT (aged 12–24 months), compared to control group (mean, *SD*)

	SCT	Control	*p‐*value	Group differences	Effect size (*η*_*p*_^2^)	Correlation with age within the SCT group
Early social communicative domains	*n =* 33	*n =* 30				
Initiating Joint Attention	17.30 (9.68)	18.80 (9.35)	0.536	SCT = control		*r* = 0.169, *p* = 0.346
Initiating Behavioral Requests	13.88 (7.81)	18.67 (10.59)	0.044	SCT < control	0.07	*r* = 0.407, *p* = 0.019
Initiating Social Interactions	0.45 (0.62)	0.63 (0.67)	0.274	SCT = control		*r* = 0.225, *p* = 0.207
Responding to Joint Attention	0.65 (0.31)	0.85 (0.19)	0.004	SCT < control	0.13	*r* = 0.487, *p* = 0.004
Responding to Behavioral Requests	16.27 (8.20)	22.47 (5.63)	0.001	SCT < control	0.17	*r* = −0.051, *p* = 0.780
Responding to Social Interactions	11.61 (6.30)	14.53 (6.87)	0.083	SCT = control	0.05	*r* = 0.454, *p* = 0.008

Abbreviation: SCT, sex chromosome trisomies.

#### Early social communication: SCT versus controls

Differences in early social communication between the SCT and control group were analyzed with a MANOVA, using Pillai's trace, with the six subscales of the ESCS as dependent variables. A significant difference between the SCT and control group was found (*F* (6, 56) = 3.28, *p* = 0.008; *η*_*p*_^2^ = 0.26). Post hoc ANOVA tests on the outcome variables revealed a significant group effect on three of the six subscales of the ESCS: Initiating Behavioral Requests (*p* = 0.044, *η*_*p*_^2^ = 0.07), Responding to Joint Attention (*p* = 0.004, *η*_*p*_^2^ = 0.13), and Responding to Behavioral Requests (*p* = 0.001, *η*_*p*_^2^ = 0.17). The multivariate significant difference between groups remained even when cognitive and language level is added as covariate (*F* (6, 53) = 1.05, *p* = 0.043, *η*_*p*_^2^ = 0.21). These results indicate more difficulties in early social communicative behaviors in 1‐year‐old children with SCT compared to the control group, with medium effect sizes. Descriptive statistics and post hoc effects can be found in Table 2.

#### Eye contact: SCT versus controls

**FIGURE 2:**
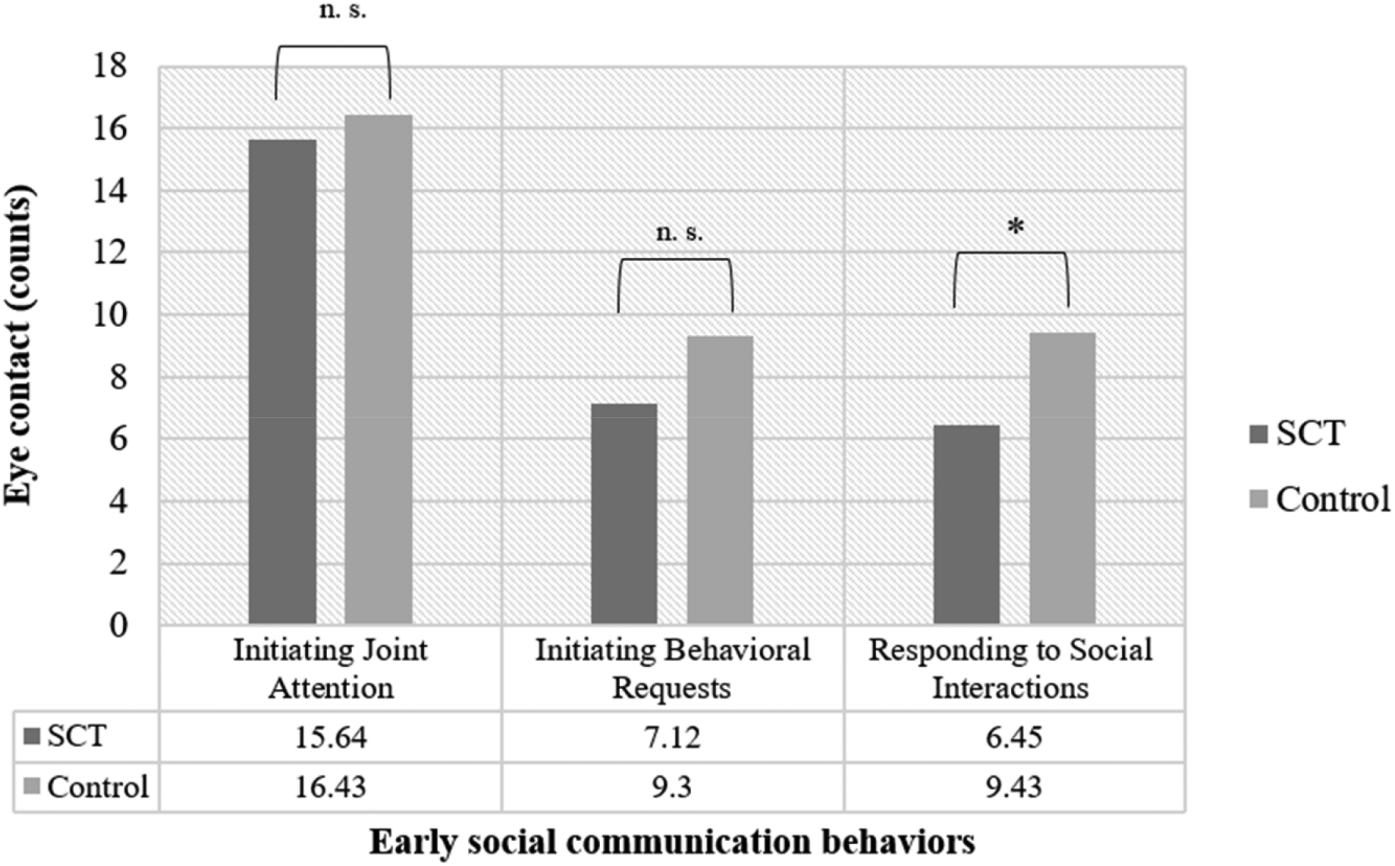
Eye contact for the SCT and control group. *p <0.05, n.s., nonsignificant; SCT, sex chromosome trisomies

Following the procedures described by Mundy (2003/2013), number of eye contact was coded under three of the six social communicative domains (Initiating Joint Attention, Initiating Behavioral Requests, and Responding to Social Interactions). Differences in eye contact between the SCT and control group were analyzed using three independent *t* tests. A significant difference is found between the SCT and control group for eye contact during Responding to Social Interactions (*t* (61) = −2.55, *p* = 0.013, Cohen's *d* = 0.64), indicating less frequent eye contact in the SCT group, compared to controls. No differences were found between the SCT and control group for eye contact during initiating of social communication: Initiating Joint Attention (*t* (61) = −3.44, *p* = 0.732) and Initiating Behavioral Requests (*t* (61) = −1.49, *p* = 0.140). See Figure 2 for exact means.

### 3.4 Social emotional development: SCT versus controls

Within the SCT group, social emotional functioning was positively correlated with age (*r* = 0.810, *p* <0.001). An independent *t* test with Bayley SE raw score as dependent variables revealed a difference in overall social emotional functioning between the SCT (*M* = 81.68, *SD* = 16.05), and the control group (*M* = 93.32, *SD* = 13.71; *t* (63) = −3.13, *p* = 0.003, Cohen's *d* = 0.78). On average, parents reported that children with SCT have more social emotional difficulties compared to the control group, with a medium effect size. The significant difference between groups remained, even when cognitive and language level is added as covariate (*F* (1, 60) = 12.41, *p* = 0.001, *η*_*p*_^2^ = 0.17). When evaluating scores normalized for age, for overall social emotional functioning, 56% of the SCT group scored in the average range, 38% scored in the borderline/monitoring range, and 6% in the extremely low/at risk range.

### 3.5 Association between early social communication and overall social emotional development

To explore the associations between early social communication behaviors and daily life social–emotional development within the SCT group, Pearson's correlations were calculated. Significant positive correlations were found between three early social communicative domains and social emotional development (initiating behavioral requests, *r* = 0.453; responding to joint attention, *r* = 0.514; and responding to social interactions, *r* = 0.346). See Table 3 for *r* and *p* values for all variables.

**TABLE 3:** Correlations between early social communication and social emotional development in the SCT group

Early social communicative domains	*r*‐value	*p*‐value
Initiating Joint Attention	0.264	0.138
Initiating Behavioral Requests	0.453	0.008
Initiating Social Interactions	0.269	0.130
Responding to Joint Attention	0.514	0.002
Responding to Behavioral Requests	−0.114	0.528
Responding to Social Interactions	0.346	0.049

Abbreviation: SCT, sex chromosome trisomy.

### 3.6 The role of specific karyotype (XXX, XXY, XYY)

In order to investigate whether specific karyotype‐subtype (XXX, XXY, XYY) was predictive of social emotional abilities and early social communication, linear regressions with dummy coding were carried out with social functioning as dependent variables. No significant predictive effects of karyotype‐subtype were found. See Table 4 for exact *F* and *p* values.

**TABLE 4:** Regression models of predictive karyotype effect on social functioning (*F* (df), *p*‐value)

	Karyotype (XXX, XXY, XYY)
ESCS	*n =* 33
Initiating Joint Attention	*F* (2, 30) = 0.69, *p* = 0.509
Initiating Behavioral Requests	*F* (2, 30) = 1.33, *p* = 0.281
Initiating Social Interactions	*F* (2, 30) = 1.33, *p* = 0.281
Responding to Joint Attention	*F* (2, 30) = 0.14, *p* = 0.868
Responding to Behavioral Requests	*F* (2, 30) = 0.74, *p* = 0.487
Responding to Social Interactions	*F* (2, 30) = 0.42, *p* = 0.663
Bayley SE	*n =* 34
Social Emotional Behavior	*F* (2, 31) = 0.00, *p* >0.999

Abbreviation: ESCS, Early Social Communication Scales.

### 3.7 The role of recruitment strategy

Within the SCT group we tested whether recruitment strategy was predictive of social emotion abilities and early social communication. We used a linear regression with dummy coding for the three recruitment strategies (A, prospective follow‐up; B, information seeking parents; and C, clinically referred cases group), and social functioning as dependent variables. No significant predictive effects of recruitment strategy were found on the Bayley SE and for five subscales of the ESCS. Only for the subscale Responding to Social Interactions of the ESCS, a predictive effect of recruitment strategy was found. See Table 5 for exact *F* and *p* values.

**TABLE 5:** Regression models of predictive recruitment strategy effect on social functioning (*F* (df), *p*‐value)

	Recruitment strategy (prospective follow‐up, information seeking parents, and clinically referred cases)
ESCS	*n =* 33
Initiating Joint Attention	*F* (2, 30) = 1.52, *p* = 0.236
Initiating Behavioral Requests	*F* (2, 30) = 0.53, *p* = 0.597
Initiating Social Interactions	*F* (2, 30) = 0.44, *p* = 0.646
Responding to Joint Attention	*F* (2, 30) = 0.98, *p* = 0.387
Responding to Behavioral Requests	*F* (2, 30) = 0.83, *p* = 0.444
Responding to Social Interactions	*F* (2, 30) = 3.77, *p* = 0.035
Bayley SE	*n =* 34
Social Emotional Behavior	*F* (2, 31) = 1.04, *p* = 0.367

Abbreviation: ESCS, Early Social Communication Scales.

## 4 DISCUSSION

The aim of the current study is to investigate the early impact of SCT (XXX, XXY, XYY) on both responding and initiating early social communicative behaviors and parent‐reported daily life social emotional functioning of young children, aged 12–24 months. On average, children with SCT show reduced frequency of eye contact in responding to social communication and appeared to have vulnerabilities in early social communication and social emotional development. These findings were independent of global cognitive and language functioning. No karyotype‐specific differences (XXX, XXY, XYY) were found.

We used systematic behavior observations to explore social communication behaviors in 1‐year‐old children with SCT when they were actually exposed to social interactions in a structured play situation. These structured observations show that, on average, 12–24‐month‐old children with SCT display lower frequency of eye contact in responding to social communication, as compared to their peers. Overall the systematic behavior observations indicate that 1–2‐year‐old children with SCT have difficulties with early social communication, expressed on the domains of joint attention, nonverbal expressions of desires and/or beliefs, and reciprocal social interactions. These findings are in line with results on parental reports of domains of problem behavior operationalized with the Child Behavioral Checklist (Urbanus, Swaab, et al., 2020), showing that social emotional differences may already be present from the age of 1 year, and independent of karyotype (XXX, XXY, and XYY).

Next, in exploring detailed aspects of early social communication in young children with SCT, it is found that children with SCT seem to have more difficulties in the way they respond to social initiatives of others, compared to how they initiate social communication. To illustrate, the ability of young children with SCT to initiate joint attention is intact, but they have difficulties with following the direction of gaze and gestures of others, that is, responding to the invitations for joint attention of others. A similar pattern is found in the way young children in SCT are involved in reciprocal social interactions: young children with SCT are able to initiate interactions, but responding to others' invitations of social interactions is impaired. Difficulties with responding to social interactions are focused on vulnerabilities in making eye contact while responding to social interaction invites: although no overall difference between the SCT and control group was found on the domain of responding to social interactions, it was found that children with SCT make eye contact less often than their typically developing peers. These results suggest that 1–2‐year‐old children with SCT do not deliberately use eye contact to respond to invitations for social interaction, but are capable of shaping a response behaviorally. With regard to the communication of desires and beliefs, young children with SCT have difficulties with both communicating their own desires and beliefs and to respond to desires of a social partner. Although speculative, the global finding that on average social communication is more affected in the way 1–2‐year‐old children with SCT *respond* to social communication of others compared to how they *initiate* social communication is in line with a reported decreased attention to focus on key social information such as faces and eyes (as for example found in children with SCT and adults with XXY; Bouw et al., 2021; Van Rijn, 2015). Existing evidence also show that children and adults with XXX and XXY have difficulties with understanding, following and labeling social cues of others as for example social gaze directions, and recognition of facial affects (Samango‐Sprouse et al., 2018). From the age of 4 years old difficulties with generating adequate social behavioral responses to a social partner was reported in XXX, XXY, and XYY (see for a review: Van Rijn, 2019). However, independent of the mechanisms that leads to difficulties with social communication as found in the current study (either difficulties with perceiving, processing, or reacting to social information), the difficulties with social communication early in life of children with SCT may lead to difficulties with forming reciprocal social contact with others.

The results from the current study regarding the early impact of SCT on social emotional development are in line with this suggestion, as they show that social communication deficits in young children with SCT extend to broad social emotional functioning in daily life settings as reported by parents. It was found that the children with SCT had more difficulties showing some typical socio‐emotional behaviors, such as a limited ability to search proximity of the caregiver, and with showing imitations of familiar make‐believe play and emotions in a back and forth way. In this study, the difference on social emotional development between children with SCT and their peers remain significant after controlling for general cognitive development. With regard to social emotional development, the proportion of children with SCT that scored in the borderline (38%) or extremely low range (6%) reveal that these social emotional impairments are present in a substantial subset of 1‐year‐old children with SCT.

The age range of the participants in this study (12–24 months) encompasses a period of development where many milestones are achieved each month. Within the SCT group, positive associations were found between age and social emotional development and between age and some of the domains of social communication (i.e., Initiating Behavioral Requests, Responding to Joint Attention, Responding to Social Interactions). These results suggest an age‐related development of social emotional and social communicative abilities in older children with SCT. Further investigations are needed to study longitudinal pathways of individual children to study early social developmental milestones even more in depth.

Even though social–emotional and communication vulnerabilities are found, our findings of intact ability to initiate joint attention and initiate social interaction tentatively suggest that on average 12–24‐month old children with SCT have a motivation to spontaneously seek and share affective experiences with others. Earlier studies on parent‐report social motivation of children with SCT from the age of 4 years found mixed results: depending on age and included karyotypes studied, no impact of SCT on social motivation was found in 4–18 years old with XXY or XYY by Cordeiro et al. (2012), whereas two other studies did find an impact of SCT on social motivation in 9–18 years old with XXX or XXY (Van Rijn et al., 2014) and 6–21 years old with XXY (Tartaglia, Cordeiro, et al., 2010). However, it should be noted that these studies utilized different parents report measures and had differences in sample ascertainment. Follow‐up studies are needed to explore the developing pathways of motivation for social communication and underlying social cognitive and motivational mechanisms in young children with SCT from the first years of life into childhood. This is especially important in order to find targets of early/preventive support and intervention, based on the idea that early intact motivation for social contact should be preserved over the course of development.

The impact of SCT on social communication and social emotional development early in life as found in this study has implications for our understanding of brain‐behavior pathways leading to these difficulties. The biological predisposition of SCT allows us to study early social development of a homogeneous group of children, which may serve as “high risk” group when it comes to neurobehavioral social development. Raznahan et al. (2016) found that in a group of participants with sex chromosome aneuploidies aged 5–25 years old, the X‐ and Y‐chromosome congruently impact the functionality of cortical areas that support adaptive socio‐emotional functioning and social communication (e.g., medial prefrontal cortex, anterior cingulate, and superior temporal sulcus). The difficulties we detect on the social domain in 12–24‐month‐old children with SCT may reflect an impact of SCT on the maturation of an integrated social brain network already from infancy on, as these social difficulties are behavioral expressions of an impaired maturation of cortical brain networks that underlie social development (Johnson et al., 2005). The finding that there are no differences between karyotype‐subtypes (XXX, XXY, XYY) on social emotional and communicative difficulties is in line with a convergent influence of the extra X‐ and Y‐chromosome on social brain maturation and associated social behavioral functioning. However, it was shown that the social behavioral profile in boys with 47,XYY is more vulnerable as compared to girls and boys with an extra X chromosome, which is illustrated by a higher risk for ASD (Cordeiro et al., 2012; Ross et al., 2012; Tartaglia et al., 2017). This more pronounced vulnerability in the XYY group was not found in the current study. However, our sample size in the XYY group was small. These findings call for more research into the nature of early social cognitive and behavioral developmental pathways in boys with 47,XYY leading to the more pronounced reported social behavioral difficulties from school age on, as compared to boys and girls with an extra X chromosome.

From a developmental perspective, it is known that vulnerabilities in early social communication and emotional functioning have a high impact on further social (cognitive) development (Mundy & Newell, 2007). To illustrate, in responding to invitations of joint attention young children show their understanding of bids to engage in another's attention. A decreased tendency to respond to these invitations may contribute to an impaired development of learning to decode and reason about mental states of others (i.e., theory of mind; Sodian & Kristen‐Antonow, 2015). Also, difficulties with social communication (e.g., less frequent eye contacts and difficulties with joint attention) early in life are reported in children with neurobehavioral disorders, such as autism spectrum disorders (ASD; see for a review: Sivaraman et al., 2020). Vulnerabilities for social emotional problems and difficulty with social communication early in life may thus be markers for difficulties in broad social functioning later on in life, and signs of potential comorbid conditions such as ASD, which is shown to be significantly elevated in SCT as compared to the typical population (Van Rijn, 2019). In particular, the ability to response to joint attention has been frequently reported in the literature as an early marker for ASD (Mundy, 2017) and therefore also serves as an important target to monitor during early development in young children with SCT.

Deficits in early social communication may not only be associated with impairments in subsequent social development, but also with an altered language acquisition, speech delay and behavioral problems during early development (Pickard & Ingersoll, 2015; Schietecatte et al., 2012). It is found in nonclinical samples that early social skills are associated with school readiness and early school success (Ziv, 2013). Given the association of early social emotional and communication with impairments on subsequent development across domains, the outcomes of the current study may have considerable clinical implications. It is important to closely monitor patterns of social communication in young children from an early age on, as difficulties in early social communication (e.g., responding to joint attention, making eye contact) may be key indicators of compromised cross‐domain development. Close monitoring of vulnerable children with SCT and if necessary early support and tailored intervention may positively influence social development through childhood.

The current study has both strengths and limitations. Strengths include the structured observations of social interactions, which allow us to measure observed social communicative behaviors of 1‐year‐old children with SCT in interaction with a social partner. Second, the study sample consisted mainly (94%) of children that were prospectively studied after a prenatal diagnosis of SCT which suggest that our findings are highly representative for the group of diagnosed children. In this study, social outcomes were largely not dependent on recruitment strategy (i.e., prospective follow‐up group, information seeking parents group, or clinically referred cases group), which suggests that on average our findings are representative for this group of diagnosed children. However, it should be taken into account that the sample sizes were small and vary between the recruitment groups. Similar, although no predictive effect of karyotype‐subtype was found on social outcomes, it is important to consider the small sample sizes and the small number of participants in the XXX and XYY group which calls for a careful interpretation of the nonsignificant differences between karyotype‐subtype. Additional studies with larger sample sizes are needed to investigate the individual X‐ and Y‐chromosome influences on social functioning very early in life of children with SCT. Next, parents of children with SCT are aware of their child's diagnoses of SCT. As a consequence, they may rate the items of the Bayley SE questionnaire differently compared to parents of children in the control group, as parents of children with SCT are more or less aware what to expect in terms of their child's developmental outcome. This may have biased the outcome of parent reports.

The findings of this study deserve further investigation of the longitudinal effects of early difficulties in social competence on social cognitive and behavioral outcomes and related neurodevelopmental psychopathology in children with SCT. These longitudinal effects will be further investigated in this population with prospective follow‐up. As it was beyond the scope of this study to investigate the role of testosterone treatment in boys with 47,XXY, future studies with an applicable design (i.e., Randomized Controlled Trials) are needed to explore the influence of these variables in association with social behavioral functioning in young children with SCT.

## 5 CONCLUSION

In summary, the results of this study show that already very early in development, that is, at the age of 1 year, children with SCT have vulnerabilities in social emotional functioning and the early ability to socially communicate with others. These results suggest an impact of SCT on social functioning very early in life, and indicate that social difficulties found later in life of individuals with SCT are anchored in early social brain development. The study of this genetic “high risk” group, characterized by an extra X‐ or Y‐chromosome may provide unique insights in the predictive value of early social communication deficits for neurobehavioral problems, learning impairments and mental health problems later in life. The findings of the current study advocate for close monitoring and early (preventive) support targeted at the earliest stages of social communication, with the aim to support social development of children with SCT from infancy on.

## CONFLICT OF INTEREST

The authors declare that there is no conflict of interest that could be perceived as prejudicing the impartiality of the research reported.

## AUTHOR CONTRIBUTIONS

**Nienke Bouw:** Design; data collection; acquisition of data; analysis; interpretation of the data; drafting. **Hanna Swaab:** Conception; design; final approval of the manuscript. **Nicole Tartaglia:** Data collection; final approval of the manuscript. **Anna C. Jansen:** Data collection; final approval of the manuscript. **Sophie van Rijn:** Conception; design; interpretation of the data; final approval of the manuscript.

## Data Availability

The data that support the findings of this study are available from the corresponding author upon reasonable request.
